# Supplementary material for: The Differential DNA Hypermethylation Patterns of microRNA-137 and microRNA-342 Locus in Early Colorectal Lesions and Tumours
Source: Biomolecules. 2019 Sep 21;9(10):519. doi: 10.3390/biom9100519 (PMC6843302; doi:10.3390/biom9100519)
Supplement: Supplementary file 1 [file biomolecules-09-00519-s001.pdf]

Supplementary Data

Table S1: Frequencies of demographic, risk factor, life-style features.

Demographic feature	Conditions	Frequency (% and number)
Ethnicity	Fars	57.6% (34)
	Tork	27.1% (16)
	Kord	8.5% (5)
	Lor	6.8% (4)
Education	Illiterates and primarily educated	40.7% (24)
	Diplomas	27.1% (16)
	Academic	32.2% (19)
Occupation	More prone to cancer-related chemicals	11.9%(7)
	Less prone to cancer-related chemicals	88.1%(52)
Fast food intake	Seldom	23.7% (14)
	Often	76/3% (46)

Table S2: Frequencies of symptom-related features

Symptom	Conditions	Frequency (% and number)
Chronic constipation	Yes	40.7% (24)
	No	59.3% (35)
Chronic diarrhea	Yes	23.7% (14)
	No	76.3% (45)
Rectal bleeding	Seen	32.2% (19)
	Not seen	67.8% (40)
Fatigue	Seen	30.5% (18)
	Not seen	69.5% (41)
Rapid weight reduction	Seen	18.6% (11)
	Not seen	81.4% (48)
Continual abdominal pain	Seen	40.7% (24)
	Not seen	59.3% (35)
Anaemia	Positive	13.6% (8)
	Negative	86.4% (51)

Table S3: Chi-Square analysis results of possible correlations between miRNA-137 promoter methylation status and clinical- pathological features

**Feature**	**Conditions**	**frequencies**	**miRNA-137M**	**miRNA-137U**	**p-value**	**Fisher`s exact test**
Pathology	Adenoma<br>Serrated	41<br>18	8(%19.5)<br>3(%16.7)	33(%80.5)<br>15(%83.3)	0.796	1.000
Dysplasia	Low grade<br>Moderate/high grade	35<br>24	3(%8.6)<br>8(%33.7)	32(%91.4)<br>16(%66.3)	0.016	.037
Anatomic site in colon	Rectum<br>Colon	23<br>36	6(%26.1)<br>5(%13.9)	17(%73.9)<br>31(%86.1)	0.241	.310
Type	Polyp<br>Tumor	51<br>8	8(%15.7)<br>3(%37.5)	43(%84.3)<br>5(62.5)	.141	.160
Size	1-10mm<br>>10mm	23<br>36	3(%13)<br>8(%22.2)	20(%87)<br>28(%77.8)	.377	.502
Number	1<br>>1	35<br>24	7(%20)<br>4(%16.7)	28(%80)<br>20(%83.3)	.747	1.000
Sex	Female<br>Male	30<br>29	1(%3.3)<br>10(%34.5)	29(%96.7)<br>19(%65.5)	.002	.002
Age	<50 yrs<br>>50 yrs	19<br>40	3(%15.8)<br>8(%20)	16(%84.2)<br>32(%80)	.698	1.000
BMI	<25<br>25 and more	16<br>43	2(%12.5)<br>9(%20.9)	14(%87.5)<br>34(%79.1)	.460	.710
Ethnicity	Fars<br>Others	34<br>25	6(%17.6)<br>5(%20)	28(%82.4)<br>20(%80)	.819	1.000
Education	Academic<br>Illiterates and primarily educated	24<br>35	5(%20.8)<br>6(%17.1)	19(%79.2)<br>29(%82.9)	.721	.745
Occupation	less prone More Prone to cancer-related chemicals<br>Less prone to cancer-related chemicals	52<br>7	10(%19.2)<br>1(%14.3)	42(%80.8)<br>6(%85.7)	.752	1.000
Family history	Positive<br>Negative	17<br>42	8(%47.1)<br>3(%7.1)	9(%52.9)<br>39(%92.9)	.000	.001

Diabetes	Positive<br>Negative	6<br>53	1(%16.7)<br>10(%18.9)	5(%83.3)<br>43(%81.1)	.896	1.000
Blood pressure	Positive<br>Negative	10<br>49	1(%10)<br>10(%20.4)	9(%90)<br>39(%79.6)	.441	.670
NSAID consumption	Positive<br>Negative	12<br>47	2(%16.7)<br>9(%19.1)	10(%83.3)<br>38(%80.9)	.844	1.000
Smoking	Positive<br>Negative	14<br>45	2(%14.3)<br>9(%20)	12(%85.7)<br>36(%80)	.632	1.000
Alcohol consumption	Positive<br>Negative	9<br>50	5(%55.6)<br>5(%12)	4(%44.4)<br>44(%88)	.002	.008
Addiction	Positive<br>Negative	2<br>57	2(%100)<br>9(%15.8)	0(%0)<br>48(%84.2)	0.003	.032
Regular exercise	Positive<br>Negative	32<br>27	6(%18.8)<br>5(%18.5)	26(%81.3)<br>22(%81.5)	.982	1.000
Vegetable/fruit consumption	Considerate<br>Insufficient	30<br>29	5(%16.7)<br>6(%20.7)	25(%83.3)<br>23(%79.3)	.692	.748
Red meat consumption	Considerate<br>Inappropriate	22<br>37	6(%27.3)<br>5(%13.5)	16(%72.7)<br>32(%86.5)	.189	.300
Gassy drink consumption	Moderate intake<br>Too much	14<br>45	3(%21.4)<br>8(%17.8)	11(%78.6)<br>37(%82.2)	.759	.712
Chronic constipation	Yes<br>No	24<br>35	5(%20.8)<br>6(%17.1)	19(%79.2)<br>29(%82.9)	.721	.745
Chronic diarrhea	Yes<br>No	14<br>45	3(%21.4)<br>8(%17.8)	11(%78.6)<br>37(%82.2)	.759	.721
Rectal bleeding	Seen<br>Not seen	19<br>40	2(%10.5)<br>9(%22.5)	17(%89.5)<br>31(%71.5)	.270	.476
Fatigue	Seen<br>Not seen	18<br>41	3(%16.7)<br>8(%19.5)	15(%83.3)<br>33(%80.5)	.796	1.000
Rapid weight reduction	Seen<br>Not seen	11<br>48	2(%18.2)<br>9(%18.8)	9(%81.8)<br>39(%81.3)	.965	1.000
Abdominal pain	Seen<br>Not seen	24<br>35	4(%16.7)<br>7(%20)	20(%83.3)<br>28(%80)	.747	1.000
Anemia	Seen<br>Not seen	8<br>51	2(%25)<br>9(%17.6)	6(%75)<br>42(%82.4)	.620	.635
